# Proton-solute coupling mechanism of the maltose transporter from *Saccharomyces cerevisiae*

**DOI:** 10.1038/s41598-017-14438-1

**Published:** 2017-10-30

**Authors:** Ryan Henderson, Bert Poolman

**Affiliations:** Department of Biochemistry Groningen Biomolecular Sciences and Biotechnology Institute and Zernike Institute for Advanced Materials University of Groningen Nijenborgh 4, 9747 AG Groningen, The Netherlands

## Abstract

Mal11 catalyzes proton-coupled maltose transport across the plasma membrane of *Saccharomyces cerevisiae*. We used structure-based design of mutants and a kinetic analysis of maltose transport to determine the energy coupling mechanism of transport. We find that wildtype Mal11 is extremely well coupled and allows yeast to rapidly accumulate maltose to dangerous levels, resulting under some conditions in self-lysis. Three protonatable residues lining the central membrane-embedded cavity of Mal11 were identified as having potential roles in proton translocation. We probed the mechanistic basis for proton coupling with uphill and downhill transport assays and found that single mutants can still accumulate maltose but with a lower coupling efficiency than the wildtype. Next, we combined the individual mutations and created double and triple mutants. We found some redundancy in the functions of the acidic residues in proton coupling and that no single residue is most critical for proton coupling to maltose uptake, unlike what is usually observed in related transporters. Importantly, the triple mutants were completely uncoupled but still fully active in downhill efflux and equilibrium exchange. Together, these results depict a concerted mechanism of proton transport in Mal11 involving multiple charged residues.

## Introduction

The first step of sugar metabolism in yeast typically involves transport of the molecule into the cell. Monosaccharides like glucose, fructose, and galactose are transported by facilitated diffusion^1^, whereas disaccharides like maltose are taken up by a proton-coupled symport mechanism^2,3^. There are five known maltose-H^+^ symporters in the *MAL* family^1^. Uniquely, Mal11 catalyzes the proton-coupled symport of a broad range of substrates containing an α-glucosyl moiety including maltose, sucrose, trehalose, maltotriose, and others^2–5^. Mal11 is a member of the Sugar Porter family (TCDB 2.A.1.1) of the Major Facilitator Superfamily (MFS) (http://www.tcdb.org). While homologous transporters do exist, much of the family exhibits low sequence identity, and the defining differences between a uniporter and a symporter are not apparent from sequence information. MFS proteins catalyze the transmembrane transport of a wide range of substrates and are found across the three domains of life^6^. Most MFS transporters consist of 12 transmembrane helices (TMs) and carry out downhill facilitated diffusion of substrate or couple the uphill movement of substrate to the electrochemical gradient of a co-substrate such as H^+^ or Na^+^ in a symport or antiport mechanism^7^.

**Figure 1:**
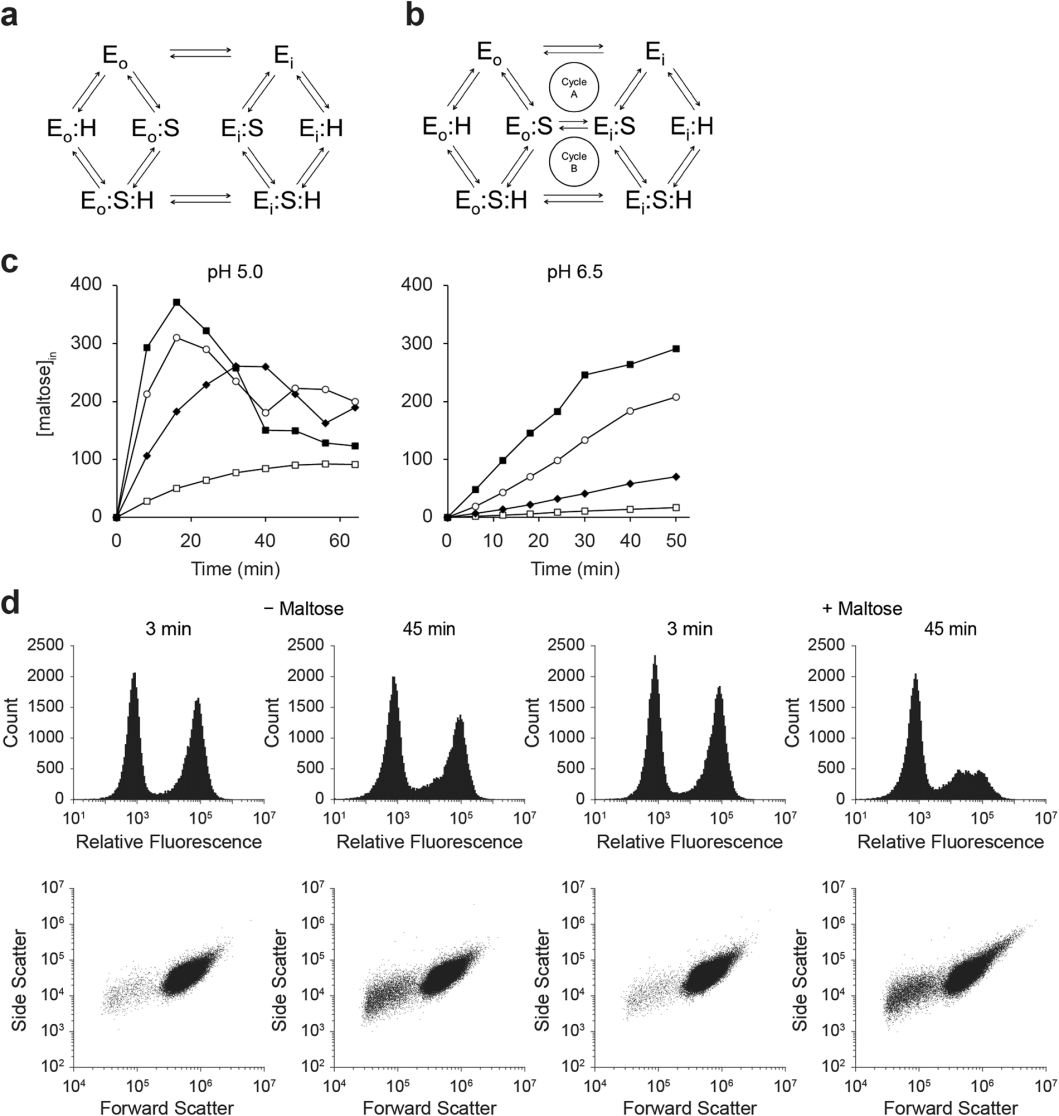
Maltose uptake by Mal11-YPet. (**a**) Kinetic scheme of a proton-coupled symporter with random binding and unbinding of substrate and proton. The outward-facing enzyme (E_o_) is available for binding either substrate, but is forbidden from switching to the inward-facing conformation (E_i_) when only one ligand is bound. Thus, transport occurs when both substrate and proton are bound via the transition between E_o_:S:H and E_i_:S:H. (**b**) A proton-coupled symporter containing an ES leak pathway in which the isomerization from out-to-in or in-to-out is only forbidden in the proton-bound state but not when only the substrate is bound. This creates two competing cycles; cycle A is favored at high pH and under conditions of high intracellular substrate concentration (substrate leak), whereas cycle B represents a substrate-dependent proton leak. (**c**) Uphill maltose transport by IMK289 cells expressing Mal11-YPet from the *GAL1* promoter of pRHA00L, washed and diluted in K-citrate-phosphate at pH 5 (left) and pH 6.5 (right). [U-^14^C]maltose was added after an initial 5 min incubation at 30 °C in the presence of 10 mM galactose. Transport was measured at 200 μM (◽); 1 mM (◆); 5 mM (⚬); and 25 mM (◾) of [U-^14^C]maltose. Data shown are representative examples of at least three repeated measurements; we do not show the error bars of the replicate measurements because the experimental conditions were not completely identical. (**d**) Flow cytometry of IMK289 cells expressing Mal11-YPet after 3 min and 45 min in the absence and presence of 25 mM of maltose; (top row) a histogram of fluorescence levels at 488 nm excitation and 533/30 nm emission filter; and (bottom row) a plot of forward and side scatter.

The canonical model of MFS symport is the alternating access mechanism, whereby binding of both substrates triggers a conformational change in the protein to alternately expose the substrate binding site(s) to the outside and inside of the cell. Importantly, this conformational change is permissible in the substrate-free state and the ternary complex (both substrates bound) but is forbidden when only one substrate is bound (Fig. 1a), as substrates or ions would otherwise leak into or out of the cell^8,9^. Recent structural studies have supported this symport model with evidence for gates that lock the transporter in an inward-facing or outward-facing conformation (reviewed in^10^). However, exceptions to this idealized view of symport have previously been uncovered through mutagenesis studies. Mutation of a single amino acid residue can significantly alter the coupling properties of a transporter, changing the apparent stoichiometry of transported substrate to co-substrate. This is caused by “leak” pathways, whereby the locked binary complex of substrate (or ion) with transporter becomes statistically more likely to unlock and thus transport one substrate down its concentration gradient in the absence of the other (Fig. 1b)^8^. Examples found through the extensive mutagenesis of the *Escherichia coli* lactose transporter LacY include mutants with proton leaks in the absence of substrate^11,12^ and mutants with substrate transport without proton transport^13^. Typically, one acidic residue plays a critical role in coupling solute and proton cotransport in secondary transporters. In LacY, this residue is Glu-325, which is required for (de)protonation of the transporter. Mutants with neutral substitutions to Glu-325 of LacY or to Glu-379 of the *Streptococcus thermophilus* lactose transporter LacS are unable to carry out transport steps involving proton translocation but can still catalyze exchange and counterflow of lactose^14,15^.

In this study, we first created a *de novo* structural model of Mal11 based on evolutionary co-variation of residues in the Sugar Porter family of MFS transporters, using the EVfold server^16,17^. We performed site-directed mutagenesis of key acidic residues present in the membrane domain of Mal11. The data indicate that the transmembrane acidic residues E120, D123, and E167 are all required for effective coupling of maltose and proton co-transport. Importantly, triple mutants of the three acidic residues are completely deficient in uphill maltose transport but retain full downhill efflux and exchange activity. Mutation of any or all of these three acidic residues introduces a substrate leak pathway into the maltose transporter. Together, these results suggest a mechanism involving at least three acidic residues to ensure proper proton coupling to maltose transport.

## Results

### Wildtype Mal11 is a well-coupled proton-maltose symporter

In ion-linked secondary transporters with a perfect coupling mechanism (Fig. 1a), solute accumulation ([solute_in_]/[solute_out_]) is expected to remain constant regardless of the concentration of solute, provided the driving force remains constant. By contrast, the presence of a substrate leak pathway (Fig. 1b) provides a means for the solute to leave the cell, an effect that becomes pronounced at high [solute_in_]. We examined uphill transport at various maltose concentrations in *S. cerevisiae* IMK289 expressing Mal11-YPet. We found that maltose accumulated steadily for over 30 min at extracellular concentrations below 1 mM. Unexpectedly, we observed loss of maltose from cells when the intracellular concentration reached approximately 400 mM (Fig. 1c). TLC analysis showed that maltose was not hydrolyzed or broken down over time (Supplementary Fig. 1a). We reasoned that a reduction in the proton motive force (pmf = Δ$\tilde{{\rm{\mu }}}$_H+_ ⁄ F = ZΔpH − ΔΨ) could explain the apparent efflux of maltose. Since the ΔpH ( = pH_in_ – pH_out_) is a component of Δ$\tilde{{\rm{\mu }}}$_H+_, we examined the intracellular (pH_in_) and extracellular pH (pH_out_) under maltose uptake conditions. We measured pH_in_ in *S. cerevisiae* IMK289 expressing the ratiometric GFP variant pHluorin as well as Mal11^18–20^. We found a maltose concentration-dependent drop in pH_in_ upon addition of the disaccharide to galactose-energized cells, which is consistent with maltose-proton symport (Supplementary Fig. 1b). However, the pH_in_ stabilized within 3 min after maltose addition and continued to decrease slowly, at a similar rate as cells to which only buffer was added. Furthermore, we found that pH_out_ was constant during maltose uptake (Supplementary Fig. 1c). At a given maltose concentration the ΔpH and thus most likely the Δp is constant and the loss of maltose cannot be explained by a change in the driving force.

We then used flow cytometry to examine the integrity of cells during maltose uptake. We observed a fluorescent and a non-fluorescent population of cells resulting from galactose-induced expression of Mal11-YPet, as has been previously reported for Gal1-GFP^21^. At pH 5 and in the presence of 25 mM maltose, there was a 51% reduction in the number of cells from the fluorescent population after 45 min of uptake, compared to a 60% loss of maltose in the transport assays (Fig. 1c,d). In the absence of maltose, there was only a 6% loss of fluorescent cells. This indicates that the cells lyse during maltose uptake, accounting for the apparent loss of maltose. We note that the cells have an enormous capacity to accumulate maltose – almost 400 mM after 15 min; either the high levels of maltose is toxic or the increased internal osmotic pressure is lethal for the cell. This behavior suggests that there is no major substrate leak pathway via Mal11, as maltose cannot passively leave the cell down its concentration gradient. This is different from what was observed for the bacterial lactose transporter LacS, which accumulated much lower levels of substrate with accumulation ratios that were strongly dependent on the substrate concentration^22^.

### Structural modeling of Mal11

**Figure 2:**
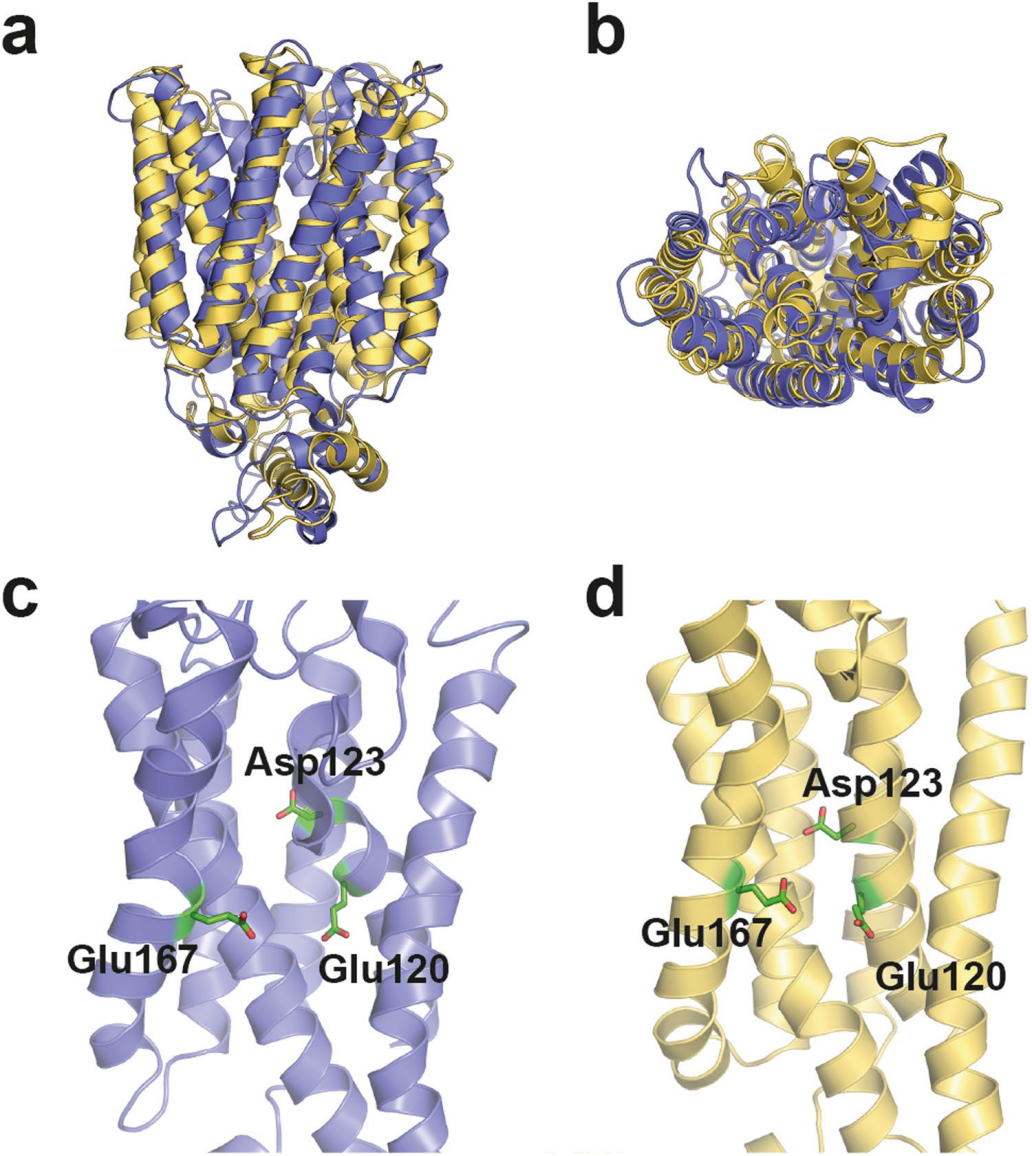
Structural modeling of Mal11. Comparison of the Mal11 structure predicted *de novo* by the EVfold server (blue), using evolutionary coupling information, to a structure based on homology modeling (yellow-orange). The overlaid structures align to an RMSD of 2.9 Å and are shown as: (**a**) a side view from within the membrane and (**b**) a top view from the extracellular side of the protein. Three acidic residues predicted to be within the transmembrane region of Mal11 are shown from a view within the membrane in (**c**) the EVfold and (**d**) the SWISS-MODEL structural predictions. The six transmembrane helices and loops from the C-half of the protein are omitted for clarity.

We sought to study the maltose-proton coupling of Mal11 in more detail and focused on the protonatable (acidic) residues in the membrane domain of the transporter. To identify the most probable proton-coupling residues of the 55 glutamates and aspartates present in the protein, we constructed a 3D structural model of the Mal11 membrane domain. We performed *de novo* structure prediction of Mal11 based on evolutionary co-variation of amino acids in MFS sugar transporters, using the EVfold server^16,17^. In brief, EVfold uses a maximum entropy analysis of the sequences of a protein family to determine evolutionary co-variation in pairs of amino acid residues at specific sequence positions. Pairs of co-evolved residues are then used as distance constraints to fold the protein of interest using the CNS software suite (see^17^ for more details). To validate the structural model with that of a known MFS transporter, we used SWISS-MODEL^23^ to perform homology modeling of Mal11. Of the MFS transporters with solved structures, we chose the bacterial xylose transporter XylE (outward-facing, partly-occluded; PDB: 4GBY^24^) as the homology modeling template because it uses a proton-symport mechanism^25^ and its sequence aligns well to Mal11 (Supplementary Table 2). Remarkably, the EVfold model aligns with the homology model with an RMSD of 2.9 Å (Fig. 2a,b). For comparison, we constructed a model of XylE using EVfold and found it aligned to the known outward-facing, partially-occluded structure of XylE with an RMSD of 4.4 Å, indicating that the Mal11 EVfold model is a plausible prediction of the actual structure. The EVfold model shows the characteristic MFS fold with 12 transmembrane helices, and the topology and tilts of the helices match closely to those of known MFS transporters^24,26,27^. The only acidic residues present in the central membrane embedded cavity are Glu-120, Asp-123, and Glu-167 (Fig. 2c,d), while the other acidic residues appear in extracellular and cytoplasmic loops or in the cytoplasmic domain. Given the proximity of Glu-120, Asp-123, and Glu-167 to each other and to the purported maltose-binding region, it seemed likely that they are involved in maltose and/or proton binding or translocation by Mal11.

### Transport properties of single Mal11 mutants

**Figure 3:**
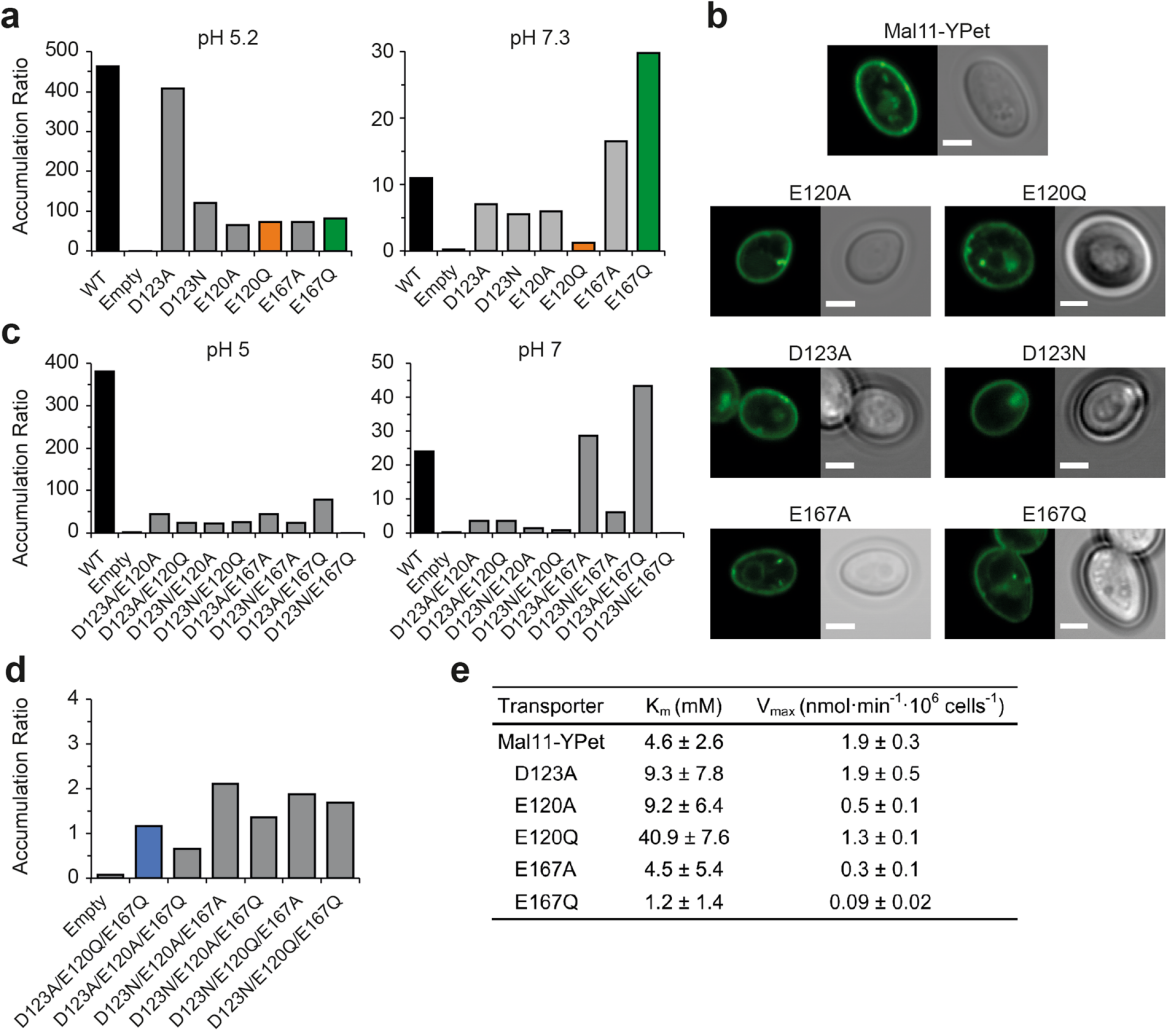
Maltose transport by Mal11 mutants. (**a**) Uphill maltose transport by IMK289 cells expressing wildtype or singly mutated Mal11-YPet from the *GAL1* promoter in K-citrate-phosphate pH 5.2 (left) and pH 7.3 (right). Cells were diluted to an OD_600_ of 4 and were incubated for 5 min at 30 °C in buffer plus 10 mM galactose, after which 1 mM [U-^14^C]maltose was added. Bars indicate the accumulation ratios ([maltose_in_]/[maltose_out_]) after 60 min of uptake. (**b**) Representative confocal fluorescence microscopy images of *S. cerevisiae* BY4742 or IMK289 cells expressing Mal11-YPet or mutants from the *GAL1* promoter on pRHA00L-based plasmids, including fluorescence (left image of each pair) and brightfield (right) images. Scale bar represents 2 μm. (**c**) Transport of 1 mM maltose by IMK289 cells expressing Mal11-YPet double mutants at pH 5 (left) and pH 7 (right) (**d**) Transport of maltose by IMK289 cells expressing Mal11 triple mutants. Conditions are the same as described in (**a**) except that cells were used at OD_600_ of 16 and the uptake after 40 min at pH 5 is shown. The data shown in (**a**), (**b**), and (**d**) are representative results of at least three repeated experiments, showing similar trends; we do not show the error bars of the replicate measurements because the experimental conditions were not completely identical. (**e**) The kinetic parameters K_m_ and V_max_ of wildtype Mal11-YPet and mutants were determined using IMK289 cells at OD_600_ of 20–27.5 in K-citrate-phosphate pH 5. 45 μL cells were equilibrated at 30 °C for 5 min before [U-^14^C]maltose was added to final concentrations ranging from 0.25 mM to 50 mM. After 2 min of incubation, the 50 μL reaction mixture was rapidly filtered as described in the Methods. Each sample was measured in triplicate and the 95% confidence range of the fit is given.

To explore the roles of the acidic residues in Mal11, we constructed neutral substitutions of the 17 acidic residues in the membrane domain of the transporter and screened the proteins for correct localization and transport capability. We initially selected *S. cerevisiae* strain BY4742 as host for the *MAL11* mutants. This strain contains intact *MAL11*, *MAL31*, *MAL12*, and *MAL31* genes but lacks a functional transcriptional regulator encoded by *MALx3*, so it cannot express the endogenous maltose metabolism proteins. Therefore, only plasmid-borne *MAL11* mutants contribute to activity. Mutations to plasma membrane proteins can result in localization problems. Without transporters in the plasma membrane, there would be no noticeable transport of maltose and no way to distinguish mutants with no activity from those without proper localization. Therefore, we constructed mutant transporters with a C-terminal YPet tag; the fluorescent protein reduced the maltose uptake rate by wildtype Mal11 to 70% compared to Mal11 with no fluorescent protein (Supplementary Fig. 2), which most likely reflects a somewhat lower level of expression of the tagged protein. Mal11-YPet localized to the periphery of the cell, as expected for a plasma membrane protein (Fig. 3b). We then screened acidic residue mutants for localization and found that most localized to the periphery, while the remaining mutants localized to the interior of the cell, likely in the cortical endoplasmic reticulum or in the vacuole (Supplementary Table 1). Importantly, all mutants of Glu-120, Asp-123, and Glu-167 localized to the cell periphery.

Maltose transport by Mal11 is driven by the electrochemical proton gradient, Δ$\tilde{{\rm{\mu }}}$_H+_^4,28^. Since BY4742 has no maltase activity, we could measure uphill maltose transport in whole cells expressing wildtype or mutants of Mal11-YPet. All mutants examined were capable of uphill maltose transport. Most mutants accumulated maltose to similar levels as wildtype Mal11-YPet except for mutants of Glu-120, Glu-167, and the Asn mutation of Asp-123 (Supplementary Table 1). These five mutants were still able to transport maltose against a concentration gradient but to much lower levels than the wildtype.

The ability of these mutants to accumulate maltose indicates the transporters are still coupled to the Δ$\tilde{{\rm{\mu }}}$_H+_ but may have an ES leak pathway, which reduces the ability to effectively accumulate solutes. For additional characterization, we transformed the six mutants of Glu-120, Glu-167, and Asp-123 into IMK289, in which all α-glucosidases and maltose transporters have been deleted and without background maltose hydrolysis and transport^29^. At pH 7.3 with 1 mM maltose, expression of Mal11-YPet in IMK289 yielded a [maltose_in_]/[maltose_out_] ratio of 11, compared to a ratio of 463 at pH 5.2 (Fig. 3a). A similar pattern was found for the six mutants, and these findings are in line with the pH dependence of maltose transport observed previously^3^. Surprisingly, both Glu-167 mutants could accumulate even more maltose than wildtype at pH 7.3 despite accumulating much less than wildtype at pH 5.2. This suggests a role for Glu-167 in mediating the *pK*_*a*_ of the proton-binding site. We note that at pH 5 the Glu-167 mutants have a similar K_m_ to the wildtype but the V_max_ is much lower (Fig. 3e). Interestingly, D123A could accumulate to 88% the level of the wildtype at pH 5.2, whereas D123N reached only 26% of wildtype accumulation.

As an additional confirmation of the dependence of transport on the Δ$\tilde{{\rm{\mu }}}$_H+_, we added the protonophore FCCP to cells expressing the wildtype or mutant transporters that had accumulated maltose and observed downhill efflux in all instances (see Supplementary Fig. 3 for an example). The diminished accumulation of the single Glu-120, Glu-167, and Asp-123 mutants may thus correspond to a change in the effective stoichiometry between maltose and proton translocation, due to the presence of a leak pathway in the transporters^8^.

### Proton-coupled maltose transport is further reduced in double mutants

To further probe the importance of the residues Glu-120, Asp-123, and Glu-167 in proton coupling of Mal11, we constructed twelve double mutants of E120A/Q, D123A/N, and E167A/Q. All double mutants containing D123A/N displayed peripheral localization, whereas we observed internal localization when Glu-120 and Glu-167 were simultaneously mutated. This points to a stability problem in transporters with Asp-123 as the only acidic residue remaining in the central cavity of Mal11. All of the double mutants with peripheral localization reached even lower accumulation ratios than the single mutants when assayed at pH 5 (Fig. 3c), except for D123N/E167Q, which had no discernable uptake. Surprisingly, mutants D123A/E167A and D123A/E167Q could accumulate more maltose than the wildtype at pH 7, whereas the other double mutants had diminished accumulation at pH 5 and 7.

### Combining all three mutations eliminates uphill maltose transport

Next, we constructed triple mutants of Glu-120, Asp-123, and Glu-167 to alanine, glutamine, or asparagine. All triple mutants localized to the cell periphery except for Mal11-E120A/D123A/E167A (Supplementary Fig. 4). Remarkably, while double mutants of Glu-120 and Glu-167 were localized to the interior of the cell, introduction of neutral substitutions at position 123 restored the proper peripheral localization of the transporters. This suggests that interactions between the three acidic residues are important for folding or stability of Mal11 in addition to proton coupling. We examined uphill transport of maltose and found that all but one of the peripherally-localized triple mutants could only equilibrate maltose ([maltose_in_]/[maltose_out_] ~ 1; Fig. 3d), demonstrating that the proton-coupled symport of maltose has been abolished. The seventh mutant, Mal11-E120Q/D123A/E167A, did not take up a discernable amount of maltose.

### Intracellular pH measurements

**Figure 4:**
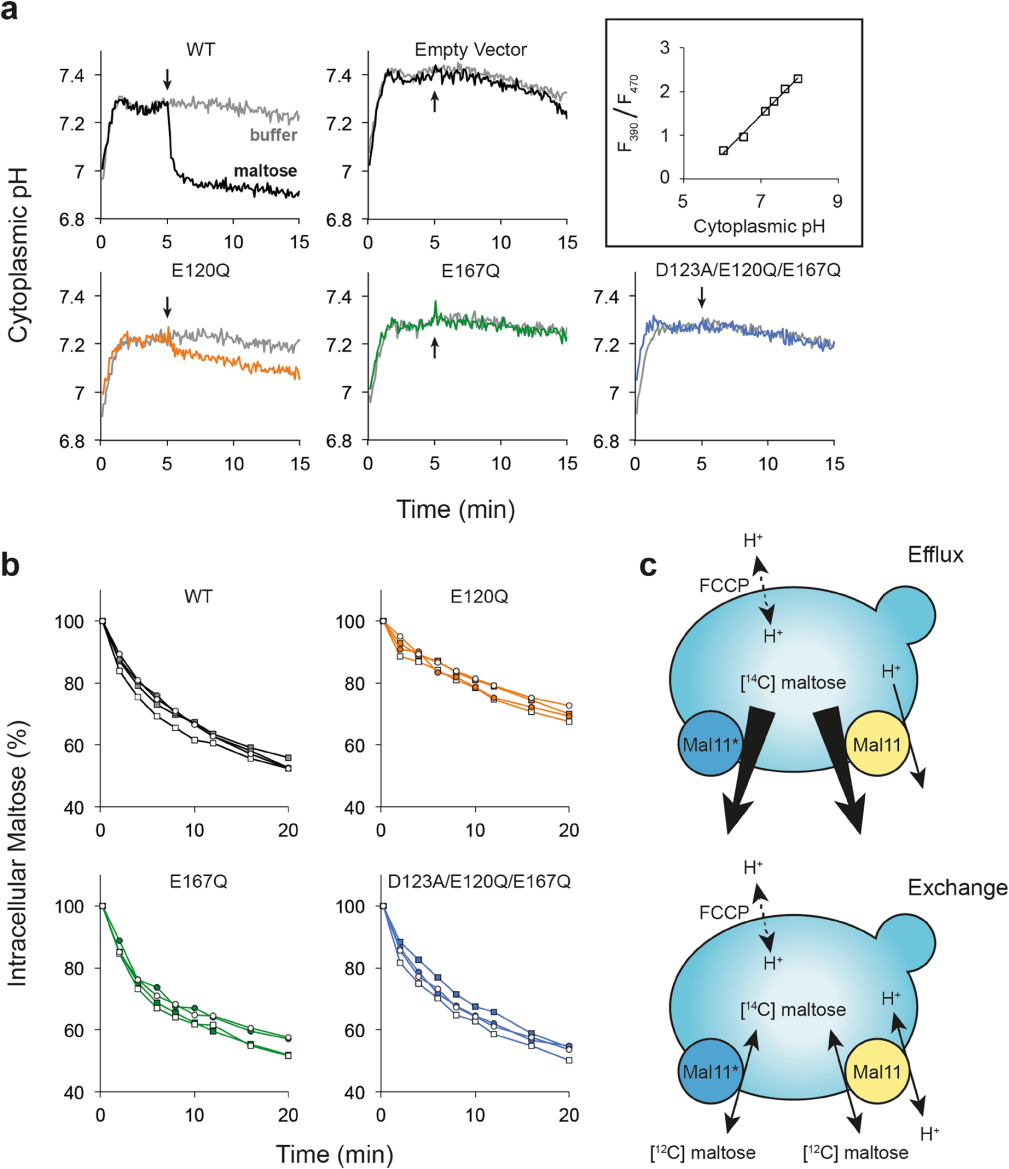
Proton cotransport and maltose efflux and exchange by wildtype Mal11 and mutant derivatives. (**a**) IMK289 cells expressing pHluorin constitutively from the *ACT1* promoter and wildtype or mutant Mal11 from the galactose-inducible *GAL1* promoter were used in the assays. A cuvette with K-citrate-phosphate pH 5 and 10 mM galactose was pre-warmed to 30 °C in the fluorescence spectrometer, and cells were added at t = 0 min to an OD_600_ of 1. The pHluorin fluorescence was determined at 390 nm and 470 nm excitation and 512 nm emission. After 5 min, 50 μL of assay buffer or maltose (to final concentration of 25 mM) was added, as indicated by the arrows. These traces are representative examples of more than three repeats that showed similar trends. (**b**) IMK289 cells expressing Mal11-YPet or mutants were pre-loaded with 10 mM [^14^C]-maltose by overnight incubation at room temperature in K-citrate-phosphate pH 5 (circles) or pH 7 (squares) in the presence of 10 μM FCCP. At t = 0 min, the overnight cells were either diluted into K-citrate-phosphate containing 10 μM FCCP (efflux, filled symbols) or K-citrate-phosphate containing 10 μM FCCP plus 10 mM maltose (equilibrium exchange, open symbols). (**c**) Diagrams showing the experimental setup of efflux (top) and exchange (bottom). In both cases, cells are preloaded with [^14^C]-maltose in the presence of FCCP. Proton-coupled transport via the wildtype transporter (Mal11) and uniport via a Mal11 triple mutant (Mal11*) are illustrated. For efflux, cells are diluted into buffer without any substrate present, permitting maltose to exit down its electrochemical gradient. Equilibrium exchange is started by dilution of cells into buffer containing an equal concentration of [^12^C]-maltose as the preloaded concentration.

We expressed pHluorin^19^ in IMK289 containing Mal11 mutants without a YPet tag. We then monitored the cytoplasmic pH of cells upon addition of either buffer or 25 mM maltose (Fig. 4a). In the strain expressing wildtype Mal11, a large drop in pH was observed when maltose was added, indicating maltose-dependent proton transport. A smaller drop (30% of wildtype) was observed for E120Q, and an even smaller one (2.4% of wildtype) for E167Q. However, there was no change in intracellular pH for the triple mutant D123A/E120Q/E167Q upon maltose addition. This demonstrates that protons are no longer co-transported with maltose, assuming the triple mutants are still capable of facilitating significant maltose transport (*vide infra*).

### Efflux and exchange of maltose by Mal11

While uphill maltose transport provides thermodynamic information about the degree of coupling in Mal11, it provides little information on the transport kinetics. Moreover, reduced transport activity may result in diminished accumulation and not necessarily reflect intrinsic (Mal11-mediated) uncoupling of solute and proton fluxes. We preloaded IMK289 cells expressing Mal11-YPet with [^14^C]-maltose in the presence of the protonophore FCCP and monitored both the efflux of maltose down its concentration gradient and the exchange of intracellular radiolabeled maltose for extracellular unlabeled maltose. To check for cell stability under these conditions, we tested the scattering properties and YPet fluorescence of the cells at regular intervals using flow cytometry. We found that the forward scatter (FSC), side scatter (SSC), relative fluorescence, and number of cells remained constant in the maltose preloading conditions for at least 21 h (Supplementary Fig. 5d–f). This indicates that the cell size and granularity were stable. Additionally, fluorescence microscopy confirmed that Mal11-YPet remained in the plasma membrane over this time period (Supplementary Fig. 5a–c).

IMK289 cells expressing Mal11-YPet and equilibrated with [^14^C]-maltose were diluted into buffer containing FCCP without (efflux) or with (exchange) added maltose, and the amount of radioactivity inside the cells was monitored over time (Fig. 4c). Figure 4b shows that the difference in efflux and equilibrium exchange catalyzed by wildtype Mal11 was relatively small at pH 5 and pH 7, suggesting that the slow step(s) of maltose transport is not reorientation of the empty carrier between outward-facing and inward-facing conformations. Since the pH doesn’t change the rate of either efflux or exchange, the rate-limiting step(s) of the transport process is pH-independent between pH 5–7.

### Maltose efflux and exchange by Mal11 mutants

The reduced accumulation by mutants could be a manifestation of decreased influx, increased efflux, or a combination of the two. We found that the rates of both efflux and exchange by wildtype, E167Q, and the triple mutant E120Q/D123A/E167Q were all similar at both pH 5 and 7, while E120Q was slower than the rest (Fig. 4b). Four additional triple mutants also catalyzed efflux and exchange of maltose at a similar rate to wildtype Mal11 (Supplementary Fig. 6). These results demonstrate that the mutations of Glu-120, Asp-123, and Glu-167, even in combination, do not significantly affect the rate-limiting step(s) of efflux or exchange by Mal11 in the absence of a proton motive force. Thus, the mutants are kinetically fully functional but are affected in the energy coupling mechanism.

## Discussion

We have characterized the proton relay network of the maltose transporter from *S. cerevisiae* and found that three acidic residues (Glu-120, Asp-123, and Glu-167) are required for full coupling and high-level accumulation of sugar. Single and double mutants reduced the ability of yeast to accumulate maltose against the concentration gradient, while triple mutants were deficient in all uphill transport activity but fully functional in transport down the concentration gradient.

We predicted the 3D structure of Mal11 using the EVfold server, which yields a structure that, unlike homology modeling, is not biased by an input model^16,17^. While it is doubtful that our model would be observed in a crystal structure, it likely represents a mixture of possible states due to the inherent conformational changes that occur during the “rocker-switch” or alternating access model of transport^9,30^. Nevertheless, the model is very good starting point for the design of mutants and the interpretation of transport data. Importantly, the transmembrane helices and many conserved residues in our EVfold model of Mal11 match in position to those found in other MFS transporters for which crystal structures are available^7,10^. One limitation of this method is that there is no reliable way to predict the presence of coordinated water molecules in the protein structures, which have been observed in the structures of MFS proteins and may have functional roles in proton-coupling and/or substrate binding^24,27,31–33^.

We found that transporters in which Glu-120, Asp-123, or Glu-167 were substituted for neutral residues accumulated less maltose than the wildtype Mal11. We propose that mutating any of the charged residues introduces a leak pathway in the transport cycle. There are a few possible leak pathways for proton-coupled symporters: ES, EH, or a combination of the two. In a secondary transporter where the substrate can also be transported in the absence of a proton (ES), transport is more likely to proceed via the ternary (protonated) complex at low pH (Fig. 1b, cycle B), whereas the binary (deprotonated) complex is preferred at high pH (Fig. 1b, cycle A)^8^. For secondary transporters with a proton leak pathway, the binary complex (EH) is favored at low pH. The reduced transport activity of all mutants with decreasing concentration of protons is indicative of an ES leak pathway. In addition, an EH leak causes a reduction in pH gradient that is (partly) reversed upon addition of substrate, and this is clearly not what we observe. Thus, by mutational analysis we have converted a solute-proton symporter into a uniporter, most likely by lowering the free energy of the ES leak pathway.

In general, unpaired charged residues and salt bridges in the hydrophobic core of transport proteins play an important role in their stability and/or catalytic activity (translocation). Two of the best-characterized proton-coupled MFS transporters are XylE^25^ and LacY^34^, and the making and breaking of salt bridges have been shown critical in translocation. Despite sharing important structural features with XylE, such as sequence motifs of the Sugar Porter family and conserved residues from the substrate-binding pocket, the key salt bridge network from XylE is not conserved in Mal11 (Supplementary Fig. 7). In XylE, there are three charged residues involved in the proton-coupling salt bridge network: Asp-27, Arg-133, and Glu-206. It has been proposed that when Asp-27 is deprotonated, it forms stable salt bridges with Arg-133 and Glu-206. These interactions are broken upon protonation of Asp-27, which is thought to trigger large-scale conformational changes. Asp-27 is a well-conserved residue in the Sugar Porter family, including in Mal11 (Asp-123). However, Arg-133 and Glu-206 of XylE are not conserved in Mal11, and we find glutamines at the corresponding positions (#214 and 284) in Mal11. We also show that Mal11 D123A has transport properties similar to that of the wildtype protein, which precludes the possibility that Mal11 has the same salt bridge network as XylE. Finally, the sequences of Mal11 and LacY cannot be aligned because the pairwise similarity/identity is not statistically significant, and thus we cannot project the salt-bridge network of LacY onto Mal11.

Asp-123 is a conserved residue among other proton-coupled transporters of the Sugar Porter family including proteins for which high-resolution crystal structures are available like XylE and GlcP_Se_. Mutating the equivalent acidic residue in some homologous transporters eliminated active sugar transport^35–38^. Indeed, most members of the GLUT family of human glucose transporters use a uniport mechanism for the monosaccharide and contain an Asn in place of the Asp found in XylE and Mal11 (Supplementary Fig. 7), which is consistent with the idea that an acidic residue in this position is critical for proton coupling. However, mutation of Asp-123 to Ala in Mal11 yielded minimal changes in accumulation of maltose, and both the Ala and Asn mutants retained proton-coupled transport activity (Fig. 3a, Supplementary Fig. 3). Furthermore, there is no basic residue near Asp-123, unlike in XylE and GlcP_Se_, where an Arg forms part of a salt bridge that is broken or rearranged upon protonation and triggers a conformational change in the transporter^35,36^. Therefore, Asp-123 may not participate directly in proton translocation in Mal11. In fact, our structural model predicts that the only transmembrane basic residue in Mal11, Arg-504, is located in helix 10 in close proximity to Glu-167. Since replacement of Glu-167 impairs proton coupling, decreases V_max_, and changes the pH-dependence of sugar transport, we speculate that this residue modulates the pK_a_ of proton binding as well as participates in a proton transfer relay during the substrate translocation cycle. Indeed, given its close proximity to Arg-504 in our model, Glu-167 is likely to have a more direct role in proton coupling than Asp-123. Finally, Glu-120 mutations were found to decrease uphill maltose transport as well (Fig. 3a). We observed that the Ala mutant has a similar K_m_ to the wildtype, but that the Gln mutant has a ten-fold higher K_m_ (Fig. 3e). Glu-120 is situated near the probable maltose-binding pocket and may coordinate the binding of a maltose to that of a proton (or *vice versa*).

Other MFS transporters exhibit a similar reliance on acidic transmembrane residues for proper proton coupling. For instance, the multidrug/proton antiporter MdfA contains two membrane-embedded acidic residues, neither of which is irreplaceable for antiport activity^39^. In fact, MdfA is dependent on the presence, but not strictly the location, of acidic residues in the central cavity^40^. Additionally, LacY has eight transmembrane charged residues, at least five of which are known to interact in a complex network of hydrogen bonds and take part in proton coupling^31,41^. Glu-325, however, is the only one that is irreplaceable for proton translocation; transport steps involving (de)protonation are blocked in Glu-325 mutants^14^. Like the LacY Glu-325 mutants, the Mal11 triple mutants could not perform uphill transport of substrate and are deficient in proton coupling. In stark contrast, whereas the LacY Glu-325 mutants had no efflux activity, the Mal1 triple mutants could perform efflux just as well as wildtype Mal11 (Supplementary Fig. 6). In fact, D123A/E120Q/E167Q could carry out both efflux and exchange of maltose at pH 5 and pH 7 at a similar rate to that of wildtype (Fig. 4b), showing that this mutant bypasses the steps involving H^+^ translocation and instead rapidly transports maltose via an ES leak pathway (Fig. 1b, cycle A).

Wildtype Mal11 is capable of accumulating maltose to exceptionally high intracellular concentrations, causing cells to eventually lyse (Fig. 1c,d). We note that an increase in internal solute (e.g. maltose) of 400 mM increases the internal osmotic pressure by about 10 atm (Δπ = ΔOsm**·**R**·**T; R = 0.082057 L**·**atm**·**K^−1^ **·**mol^−1^), from which even a rigid cell wall may not be able to protect the cell. Accordingly, only the most fluorescent cells were disrupted, thus those that expressed Mal11-YPet at high level and had the greatest transport capacity. Our observations of excessive maltose accumulation suggest a tight coupling of the maltose and proton fluxes in wildtype Mal11. A transporter with an ES-leak pathway would accumulate less since flux out of the cell increases with increasing intracellular substrate concentrations. Thus, in terms of metabolic energy conservation, a well-coupled transporter is most efficient but doesn’t allow protection under conditions of excessive solute accumulation. In growing, maltose-metabolizing yeast, the accumulation of maltose will be less because intracellular maltose hydrolase alleviates this burden. However, maltose addition to cells grown under maltose-limited conditions has been shown to cause cell death^42^. Under such growth conditions, the maltose transport capacity is much higher than in cultures with excess maltose. If transport capacity is in excess of hydrolysis activity, then accumulation of maltose will occur, which eventually will lead to cell lysis. Coupling efficiency has also been studied in the bacterial disaccharide transporter LacS, and, contrary to Mal11, this system clearly displays an ES leak pathway in the wildtype protein^8,22^. In fact, the single and double mutants of Mal11 behave similarly to wildtype LacS.

In conclusion: we propose a concerted mechanism of proton transport in Mal11, involving three anionic residues, which allow for efficient, highly coupled symport of maltose and protons. As a result, yeast can rapidly accumulate maltose to dangerous levels, resulting under some conditions in self-lysis.

## Materials and Methods

### Yeast strains and growth conditions

*S. cerevisiae* IMK289^29^, derived from CEN.PK102-3A (MATa *MALx MAL2x MAL3x leu2-112 ura3-52 MAL2-8* ^*C*^) by replacement of the maltose metabolizing loci *MALx1*, *MALx2*, *MPH2*, and *MPH3* with *loxP*, and BY4742 (MATα his3Δ1 leu2Δ0 lys2Δ0 ura3Δ0)^43^ were used to express variants of Ma11. Synthetic complete drop-out media lacking uracil (Ura) and/or leucine (Leu) were made using yeast nitrogen base without amino acids and the appropriate Kaiser amino acid drop-out supplement (both from Formedium) and either 2% (w/v) glucose (SD) or raffinose (SR). For microscopy and transport experiments, overnight cultures of yeast grown at 30 °C in selective SD media were diluted in selective SR media and induced with 0.2% (w/v) galactose the next morning, when cells were still in the exponential phase of growth. The cells were grown an additional 2 h (for BY4742 strains) or 2.5 h (for IMK289 strains) before being harvested by centrifugation.

### Plasmids and DNA manipulation

Genomic DNA was isolated from BY4742 using a commercially available plasmid purification kit (BIOKÉ, Leiden, The Netherlands). We amplified the backbone of pFB001^44^, using primer pair 5273/5274, and the *MAL11* gene from BY4742 genomic DNA, using primer pair 5271/5272. These DNA fragments were transformed into BY4742, using the lithium acetate method, and assembled by homologous recombination, resulting in pRHA00 (2μ ori, *P*_*GAL1*_ *-MAL11-TEV-YPet-T*_*CYC1*_, *URA3* marker). pRHA00L was constructed by amplifying pRHA00 without URA3, using primer pair 5437/5438, and amplifying LEU2 from pRS315^44^, using primers 5435/5436, followed by homologous recombination in BY4742. Single mutants were constructed by using PCR to amplify MAL11 from pRHA00L in two halves with 30 to 40 bp sequence overlap at the site of the mutation; the two fragments and the pRHA00L backbone were then transformed into BY4742 or IMK289 for homologous recombination. Double mutants of E120 and D123 were similarly constructed using homologous primers covering the codons for both residues at once, whereas double mutants involving E167 and triple mutants were constructed by using one of the single mutant genes as PCR template. Both double and triple mutant plasmids were assembled in IMK289; all mutants were fully sequenced and the plasmids were used to retransform IMK289. See the Supplementary Information for all plasmids (Supplementary Table 3) and primers (Supplementary Table 4) used in this study.

### Amino acid sequence alignment

Multiple sequence alignment of Mal11 with other transporters was performed using PSI/TM-Coffee^45^. Jalview was used for alignment visualization and pairwise alignment calculations^46^. Transporter sequences were found with the following UniProt accession numbers: Mal11 (P54038), XylE (P0AGF4), LacY (P02920), MelB (P02921), GLUT1 (P11166), GLUT3 (P11169).

### Mal11 structural modeling

*De novo* structure prediction using evolutionary co-variation of residue pairs was performed with the EVfold server^16,17^. The Pfam multiple sequence alignment of ~15,000 sequences from the MFS sugar transporter family (PF00083—Sugar_tr)^47^ was used for the structure prediction and was run using the default settings except that the high conservation filter threshold was set to 95%. Homology modeling of Mal11 was done using the SWISS-MODEL server^23^. A search for suitable templates yielded XylE (PDB: 4GBY)^24^ as the proton-coupled transporter most similar to Mal11 and was used to predict the structure.

### Fluorescence microscopy

Induced cells were harvested by centrifugation at 3,000 g for 5 min at 4 °C and resuspended in buffer or media. Cells were kept on ice until a sample was immobilized under a cover slip on a glass slide. Fluorescence imaging of live cells was carried out on a Zeiss LSM 710 scanning confocal microscope (Carl Zeiss MicroImaging, Jena, Germany), equipped with a C-Apochromat 40x/1.2 NA objective and a blue argon laser (488 nm). Images were captured with the focal plane at the mid-section of the cells.

### Maltose transport assays

#### Uphill transport

Induced yeast cells were harvested by centrifugation at 3,000 g for 5 min at 4 °C and washed twice by resuspending the cell pellets in 3 mL assay buffer (0.1 M potassium-phosphate (KPi) or potassium-citrate-phosphate (KCP) + 10 mM galactose) and repeating the centrifugation step. Cells were resuspended in assay buffer and kept on ice until used within 4 hours Most transport assays were performed at 30 °C using cells at OD_600_ of 4, 8 or 16, except when the kinetic parameters of transport (K_m_ and V_max_) were determined and OD_600_ of 20 to 27.5 were used. Cells were incubated at 30 °C for 5 min to increase the adenylate energy charge^48^, after which [U-^14^C]maltose (600 mCi/mmol; American Radiolabeled Chemicals, Inc.) was added to approximately 48100 Bq/mL to start the uptake reaction; the maltose concentrations varied from 0.25 mM to 50 mM. At given time intervals, 50 μL samples were added to 2 mL ice-cold KPi or KCP and rapidly filtered on cellulose-nitrate filters with 0.45 μm pores (GE-Healthcare, Little Chalfont, UK) pre-soaked in KPi or KCP plus 1 mM of maltose to block non-specific adsorption of ^14^C-maltose. Filters were washed once with 2 mL KPi or KCP and then dissolved in 2 mL scintillation solution (Emulsifier^plus^, PerkinElmer, Waltham, MA, USA). The amount of radioactivity was determined using a liquid scintillation counter (Tri-Carb 2800TR liquid scintillation analyzer, PerkinElmer). The amount of maltose in each sample was normalized to 10^6^ cells by counting cells using a flow cytometer and correcting for the fraction of fluorescent cells (see “Flow Cytometry”). We used an estimate of 60 fL internal volume per cell to calculate the intracellular maltose concentrations.

#### Efflux and equilibrium exchange

Induced yeast cells were grown and harvested as in “Uphill transport” and were washed twice in KPi or KCP containing 10 μM of the protonophore carbonyl cyanide-p-trifluoromethoxyphenyl-hydrazone (FCCP). Cell pellets were weighed and resuspended to 0.5 mg/mL wet weight in a radioactive mixture consisting of: 10 μM FCCP, [U-^14^C]maltose (final activity of ~1600 Bq/μL), and KPi or KCP at the desired pH. Resuspended cells were incubated at room temperature overnight. To start the efflux reaction, 20 μL of cells were added to 1980 μL buffer supplemented with 10 μM FCCP at 30 °C, and the loss of internal maltose was monitored over time; the radioactivity in 200 μL samples was filtered and determined as described above. Equilibrium exchange was done similarly by dilution of 20 μL of cells into 1980 μL buffer with FCCP containing 10 mM nonradioactive maltose, unless indicated otherwise in the figure legends.

### Measurement of cytosolic and extracellular pH

IMK289 bearing pYES2-*P*_*ACT1*_-pHluorin was grown, harvested, and washed as described in “Uphill transport” and resuspended to OD_600_ of 10 in assay buffer (KCP supplemented with 10 mM galactose). The cytosolic pH was calibrated and measured essentially as described previously^20^. Fluorescence measurements at 390 nm or 470 nm excitation and 512 nm emission were performed using a Jasco FP-8300 fluorescence spectrometer (Jasco, Gross-Umstadt, Germany) at 30 °C with stirring. To calibrate the cytosolic pH to the ratiometric pHluorin signal, cells were diluted to OD_600_ of 1 in assay buffers ranging from pH 6 to pH 8 plus 0.02% digitonin, incubated for 30 min to allow complete permeabilization, after which the fluorescence was measured. To observe proton cotransport, cells were diluted to OD_600_ of 1 in assay buffer in a disposable 4.5 mL plastic cuvette with four clear faces (Kartell, Noviglio, Italy) with a magnetic stir bar in the bottom and equilibrated for 5 min at 30 °C, at which point either maltose or buffer was added. On-line extracellular pH measurements were made using a ProLab1000 (SI Analytics, Weilheim, Germany) under the same conditions as described for the intracellular pH measurements.

### Thin layer chromatography

50 μL cell samples from the transport assays were mixed with 50 μL mobile phase consisting of ethyl acetate:acetic acid:methanol:water (60:15:15:10). 10 μL samples of this mixture were spotted on a rectangular piece of aluminum foil-bound silica TLC plate and resolved in a glass jar. The TLC plate was dried overnight in a fume hood. The radioactivity was detected on a phosphor storage plate and imaged after 6 days using a Typhoon 9400 scanner (GE Healthcare, Little Chalfont, UK).

### Flow cytometry

Cell samples for flow cytometry were diluted to OD_600_ of approximately 0.4 in assay buffer. 20 μL of sample were measured with an Accuri C6 flow cytometer (BD Accuri^TM^, Durham, USA). Fluorescence was detected using the flow cytometer’s built-in 488 nm laser and the “FL1” emission detector (533/30 nm).

### Data Availability

Flow cytometry data is available from the corresponding author upon reasonable request. All other data generated and analyzed during the current study are included in this article and its Supplementary Information files.

## Electronic supplementary material

Supplementary Information
