# Supplementary material for: Proton-solute coupling mechanism of the maltose transporter from Saccharomyces cerevisiae
Source: Sci Rep. 2017 Oct 30;7:14375. doi: 10.1038/s41598-017-14438-1 (PMC5662749; doi:10.1038/s41598-017-14438-1)
Supplement: Supplementary file 1 — Supplementary Information [file 41598_2017_14438_MOESM1_ESM.pdf]

Supplementary Information

Proton-solute coupling mechanism of the maltose transporter from *Saccharomyces cerevisiae*

Ryan Henderson and Bert Poolman*

Department of Biochemistry
Groningen Biomolecular Sciences and Biotechnology Institute & Zernike Institute for Advanced Materials
University of Groningen
Nijenborgh 4, 9747 AG Groningen
The Netherlands

*Corresponding author details: b.poolman@rug.nl
Tel: +31 50 3634190

Supplementary figures 1-7
Supplementary tables 1-4

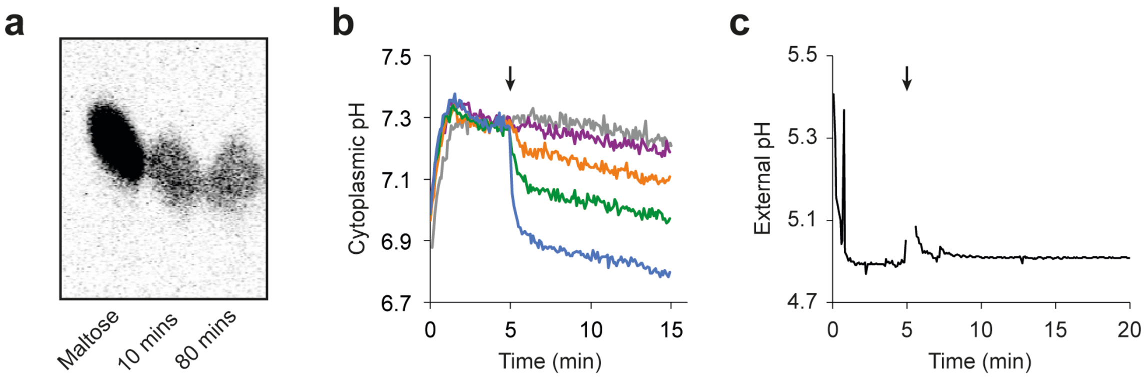

Supplementary Figure 1. *S. cerevisiae* accumulates genuine maltose. (a) Thin-Layer Chromatography was performed by sampling IMK289 cells expressing Mal11-YPet after 10 and 80 min of [^{14}C]maltose (final concentration of 1 mM) uptake. Samples were spotted on an aluminum foil-bound silica TLC plate and resolved using a mobile phase of ethyl acetate:acetic acid:methanol:water (60:15:15:10). The radioactivity was detected on a phosphor storage plate and later imaged. (b) Intracellular pH was measured, as described in the Methods section and in the legend of Fig. 4a, during uptake of maltose by IMK289 expressing pHluorin and wildtype Mal11. At 5 min (arrow), either buffer (grey) or maltose (200 µM, purple; 1 mM, orange; 5 mM, green; 25 mM, blue) was added. The experiments were performed in K-citrate-phosphate pH 5 in the presence of 10 mM galactose. (c) Extracellular pH was measured as described in the Methods section. After 5 min of incubation at 30 °C, maltose (arrow) was added to IMK289 cells expressing Mal11 and pHluorin, with pH recorded every 5 sec. Cells at an OD_{600} of 4 were used in K-citrate-phosphate pH 5.

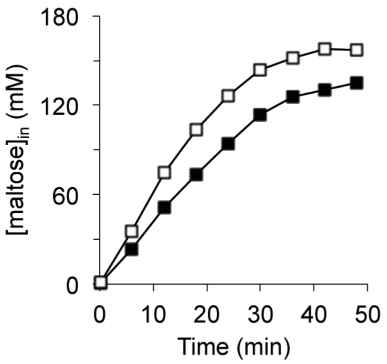

Supplementary Figure 2. Maltose uptake by Mal11 and Mal11-YPet. IMK289 cells expressing either wildtype Mal11 (□) or Mal11-YPet (■) were grown and prepared as described in the Methods section. Cells at an OD_{600} of 16 were incubated at 30 °C in the presence of 1 mM [U-^{14}C]maltose and transport was followed by taking samples at the indicated time points.

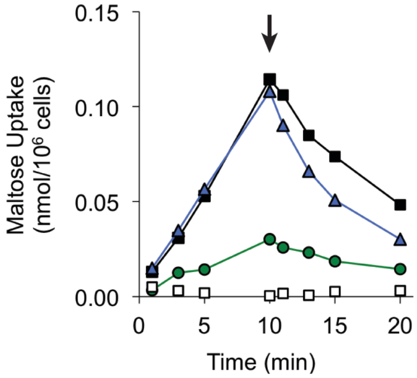

Supplementary Figure 3. FCCP dissipates the proton motive force and causes efflux of pre-accumulated maltose. BY4742 cells expressing Mal11-YPet (black squares), D123A (blue triangles), D123N (green circles), or no protein (empty plasmid; white squares) were equilibrated to 30 °C for 5 min, after which 1 mM ^{14}C-maltose was added and samples were taken to measure the transport. After 10 min of incubation with maltose, 10 μM FCCP was added (arrow) and maltose efflux was observed.

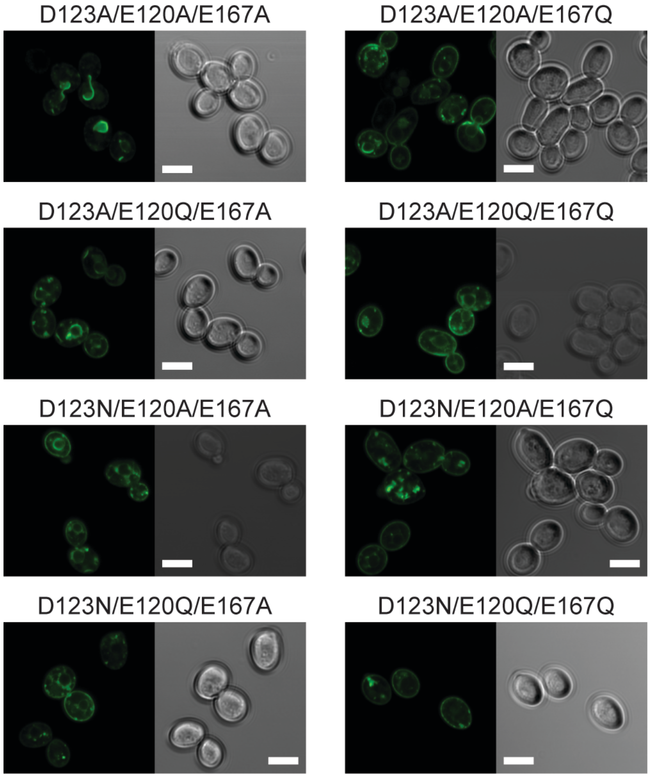

Supplementary Figure 4. Localization of Mal11-YPet triple mutants. IMK289 cells expressing Mal11-YPet triple mutants from the *GAL1* promoter of pRHA00L-based plasmids were grown and prepared as described in the Methods section. Fluorescence images obtained with 488 nm excitation (left) and brightfield (right) images are shown and the scale bar represents 2 μm.

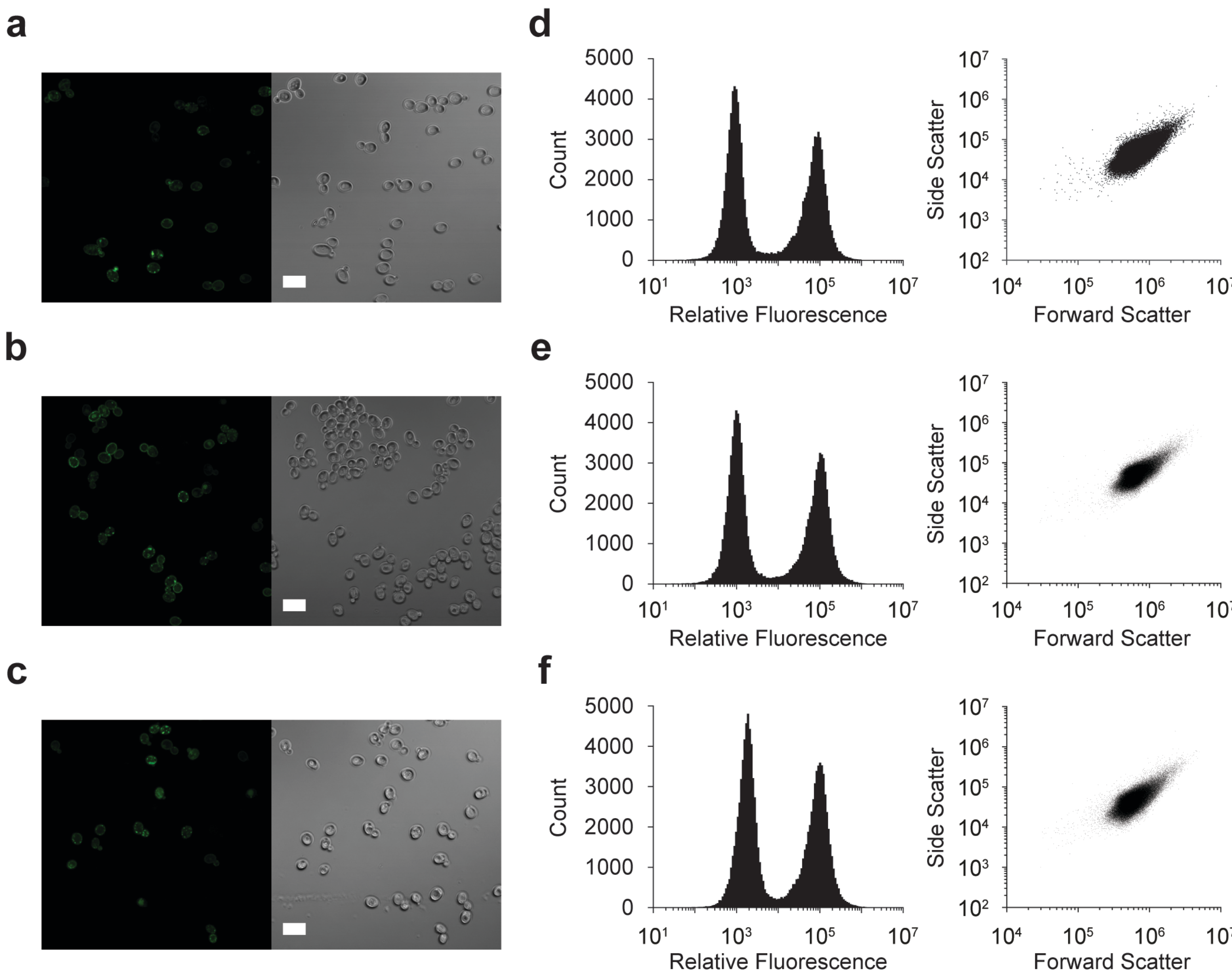

Supplementary Figure 5. FCCP treatment does not affect Mal11-YPet localization or fluorescence. IMK289 cells expressing Mal11-YPet from pRHA00L were resuspended to 0.5 mg/mL (wet weight) in K-citrate-phosphate pH 7 with 10 µM FCCP. Fluorescence microscopy and flow cytometry were performed as described in the Methods section before treatment (a,d) and after overnight incubation (21 h) at 4 °C (b, e) or 30 °C (c, f). The scale bar of the microscopy images is 2 µm. The flow cytometry results shown in d-f are the fluorescence as measured with 488 nm excitation and 533/30 nm emission filter (left) and plots of forward and side scatter (right).

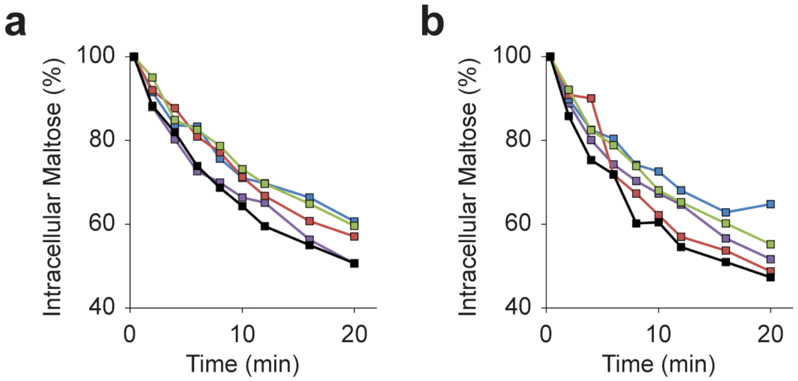

Supplementary Figure 6. Efflux and exchange of maltose by wildtype Mal11-YPet and triple mutants. (a) Efflux and (b) exchange of maltose by Mal11-YPet (black) and triple mutants D123A/E120Q/E167Q (purple), D123N/E120A/E167Q (blue), D123N/E120Q/E167A (red), and D123N/E120Q/E167Q (green) in K-phosphate pH 7 and preloaded with 1 mM ^{14}C-maltose. Cells were prepared and the experiment was carried out as described in the Methods section and in the legend of Fig. 4b, except that exchange was performed with 50 mM maltose in the buffer into which the preloaded cells were diluted at the start of the experiment.

a

```
Mal11    1 MKNIISLVSKKKAASKNEDKNISESSRDIVNQQEVFNTEDFEEGKKDSAFELDHLEFTTN  60
GLUT1    1 MEPSSKKLTGRLML----------------------------------------------  14
XylE     1 MNTQYN-SS-YIFS----------------------------------------------  12

Mal11   61 SAQLGDSDEDNENVINEMNATDDANEANSEEKSMTLKQALLKYPKAALWSILVSTTLVME 120
GLUT1   15 ------------------------------------------------AVGGAVLGSLQF  26
XylE    13 ------------------------------------------------ITLVATLGGLLF  24

Mal11  121 GYDTALLSALYA-LPV-FQRKFGT--LNGEGSYEITSQWQIGLNMCVLCGEMIGLQITTY 176
GLUT1   27 GYNTGVINAPQKVIEEFYNQTWVHRYGESILPTTLTTLWSLSVAI-FSVGGMIGSFSVGL  85
XylE    25 GYDTAVISGTVESLNTVFVAP------QNLSESAANSLLGFCVAS-ALIGCIIGGALGGY  77

Mal11  177 MVEFMGNRYTMITALGLLTAYIFIL---YY---------------CKSLAMIAVGQILSA 218
GLUT1   86 FVNRFGRRNSMLMMNLLAFVSAVLMGFSKL---------------GKSFEMLILGRFIIG 130
XylE    78 CSNRFGRRDSLKIAAVLFFISGVGSAWPELGFTSINPDNTVPVYLAGYVPEFVIYRIIGG 137

Mal11  219 IPWGCFQSLAVTYASEVCPLALRYYMTSYSNICWLFGQIFASGIMKNS---QENLGNSDL 275
GLUT1  131 VYCGLTTGFVPMYVGEVSPTALRGALGTLHQLGIVVGILIAQVFGL------DSIMGNKD 184
XylE   138 IGVGLASMLSPMYIAELAPAHIRGKLVSFNQFAIIFGQLLVYCVNYFIARSGDASWLNTD 197

Mal11  276 GYKLPFALQWIWPAPLMIGIFFAPESPWWLV-RKDRVAEARKSLSRILSGKGAEKDIQVD 334
GLUT1  185 LWPLLLSIIFIPALLQCIVLPFCPESPRFLLINRNEENRAKSVLKKLRGTADVTHD---- 240
XylE   198 GWRYMFASECIPALLFLMLLYTVPESPRWLM-SRGKQEQAEGILRKIMGNTLATQA---- 252

Mal11  335 LTLKQIELTIEKERLLASKSGSFFNCFKGVNGRRTR-LACLTWVAQNSSG-AVLLGYSTY 392
GLUT1  241 --LQEMKEESRQ--MMREKKVTILELFRSPAYRQPILIAVVLQLSQQLSGINAVFYYSTS 296
XylE   253 --VQEIKHSLDH--GRKT----GGRLLM--FGVGVIVIGVMLSIFQQFVGINVVLYYAPE 302

Mal11  393 FFERAGMATDKAFTFSLIQYCLGLAGTLCSWVISGRVGRWTILTYGLAFQMVCLFIIG-- 450
GLUT1  297 IFEKAGVQQPV--YATIGSGIVNTAFTVVSLFVVERAGRRTLHLIGLAGMAGCAILMTIA 354
XylE   303 VFKTLGASTDIALLQTIIVGVINLTFTVLAIMTVDKFGRKPLQIIGALGMAIGMFSLG-- 360

Mal11  451 GMGFGSGSSASNGAGGLLLALSFFYNAGIGAVVYCIVAEIPSAELRTKTIVLARICYNLM 510
GLUT1  355 LALLEQLPWMSYLSIVAIFGFVAFFEVGPGPIPWFIVAELFSQGPRPAAIAVAGFSNWTS 414
XylE   361 TAFYTQAP--GIVALLSMLFYVAAFAMSWGPVCWVLLSEIFPNAIRGKALAIAVAAQWLA 418

Mal11  511 AVINAILTPYMLNVS----DWNWGAKTGLYWGGFTAVTLAWVIIDLPETTGRTFSEINEL 566
GLUT1  415 NFIVGMCFQYVE-------QLC-GPYVFIIFTVLLVLFFIFTYFKVPETKGRTFDEIASG 466
XylE   419 NYFVSWTFPMMDKNSWLVAHFH-NGFSYWIYGCMGVLAALFMWKFVPETKGKTLEELEAL 477

Mal11  567 FNQGVPARKFASTVV--DPFGKGKTQHDSLADESISQSSSIKQRELNAADKC         616
GLUT1  467 FRQGGASQSDKTPEELFHPLGADSQV--------------------------         492
XylE   478 WEPETKKTQQTATL--------------------------------------         491
```

b

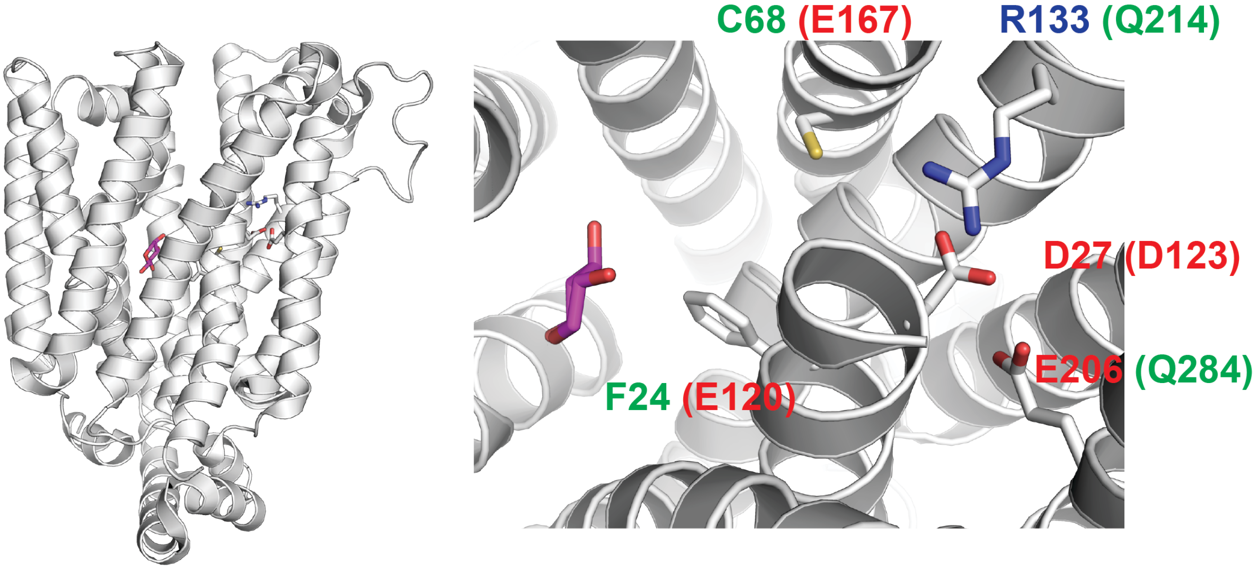

Supplementary Figure 7. The acidic residue network of Mal11 is not conserved in other sugar transporters. (a) Multiple sequence alignment of Mal11, GLUT1 (SLC2A1), and XylE, performed using PSI/TM-Coffee. Blue highlighted residues are colored according to conservation using the BLOSUM62 substitution matrix. Yellow circles indicate the charged transmembrane residues from Mal11, and green circles show the charged residues involved in the proton-coupling salt bridge network in XylE. Red underlines specify the transmembrane regions of XylE. (b) The model of XylE in the outward-occluded conformation (PDB ID: 4GBY) used to construct the homology model of Mal11 from a side view within the membrane (left) and a zoom-in from the top of the xylose and proton binding sites (right). Bound xylose is colored magenta. The residues are labeled according to XylE with the aligned Mal11 residue in parentheses and are colored green (neutral residues), red (acidic), or blue (basic).

Supplementary Table 1. Screening of Mal11 mutants for localization and uptake. BY4742 cells expressing wildtype or mutant Mal11-YPet from the *GAL1* promoter were grown and prepared as described in the legend of Fig. 1. YPet fluorescence at 488 nm was tracked with fluorescence microscopy to determine the cellular localization of each mutant: P, peripheral localization; ER, cortical endoplasmic reticulum; ND, not determined. Uphill transport of 0.1 mM or 1 mM maltose was measured for 10 minutes in K-phosphate pH 6: +, > 25 % of wildtype transport; +/-, < 25 % of wildtype transport.

Mal11 Residue	Mutant	Localization	Maltose Uptake
Wildtype	-	P	+
E120	Ala<br>Gln	P<br>P	+/-<br>+/-
D123	Ala<br>Asn	P<br>P	+<br>+/-
E146	Ala<br>Gln	P<br>P	+<br>+
E150	Ala<br>Gln	P<br>ND	+<br>ND
E167	Ala<br>Gln	P<br>P	+/-<br>+/-
E179	Ala<br>Gln	ND<br>ER	ND<br>ND
E234	Ala<br>Gln	ND<br>ER	ND<br>ND
E268	Ala<br>Gln	ER<br>P	ND<br>+
D274	Ala<br>Asn	P<br>P	+<br>+
E300	Ala<br>Gln	ND<br>ER	ND<br>ND
E395	Ala<br>Gln	P<br>P	ND<br>ND
D402	Ala<br>Asn	P<br>P	+<br>+
E489	Ala<br>Gln	ER<br>ER	ND<br>ND
E494	Ala<br>Gln	P<br>P	+<br>+
D526	Ala<br>Asn	P<br>P	+<br>+
D551	Ala<br>Asn	ND<br>P	ND<br>+
E554	Ala<br>Gln	ER<br>ER	ND<br>ND

Supplementary Table 2. Percentage identities of pairwise alignments between Mal11 and several MFS transporters. Alignments were generated using PSI/TM-Coffee and percentage identity was calculated in Jalview. For LacY and MelB, no statistically significant pairwise alignment with Mal11 could be made.

Protein	UniProt ID	Superfamily	Family	Organism	Identity with Mal11 (%)
Mal11	P54038	MFS	Sugar Porter	S. cerevisiae	-
XylE	P0AGF4	MFS	Sugar Porter	E. coli	20.74
LacY	P02920	MFS	Oligosaccharide/H+ Symporter	E. coli	No alignment
MelB	P02921	MFS	Glycoside-Pentoside-Hexuronide:Cation Symporter	E. coli	No alignment
GLUT1	P11166	MFS	Sugar Porter	H. sapiens	23.54
GLUT3	P11169	MFS	Sugar Porter	H. sapiens	23.3

Supplementary Table 3. Plasmids used in this study

Plasmid	Description	Reference
pFB001	pRS426GAL1-GFP derivative, *lyp1-TEV-YPet-his*	[44]
pRHA00L	pFB001 derivative with *LEU2* and *MAL11-TEV-YPet-his*	This study
pRHA00L0	pRHA00L derivative digested with *BssHII* to remove *GAL1-MAL11-TEV-YPet-his*	This study
pRHA37	pRHA00L derivative with *MAL11-TEV-his*	This study
pRHA06L	Mal11-D123A derivative of pRHA00L	This study
pRHA36	Mal11-D123N derivative of pRHA00L	This study
pR98	Mal11-E120A derivative of pRHA00L	This study
pR99	Mal11-E167A derivative of pRHA00L	This study
pR126	Mal11-E167Q derivative of pRHA00L	This study
pR224	pR126 derivative without *YPet*	This study
pR128	Mal11-E120Q derivative of pRHA00L	This study
pR222	pR128 derivative without *YPet*	This study
pR100	Mal11-D123A/E120A derivative of pRHA00L	This study
pR101	Mal11-D123A/E120Q derivative of pRHA00L	This study
pR102	Mal11-D123N/E120A derivative of pRHA00L	This study
pR103	Mal11-D123N/E120Q derivative of pRHA00L	This study
pR104	Mal11-D123A/E167A derivative of pRHA00L	This study
pR105	Mal11-D123N/E167A derivative of pRHA00L	This study
pR106	Mal11-E120A/E167A derivative of pRHA00L	This study
pR107	Mal11-E120Q/E167A derivative of pRHA00L	This study
pR108	Mal11-D123A/E167Q derivative of pRHA00L	This study
pR109	Mal11-D123N/E167Q derivative of pRHA00L	This study
pR110	Mal11-E120A/E167Q derivative of pRHA00L	This study
pR111	Mal11-E120Q/E167Q derivative of pRHA00L	This study
pR112	Mal11-D123A/E120Q/E167A derivative of pRHA00L	This study
pR113	Mal11-D123A/E120Q/E167Q derivative of pRHA00L	This study
pR226	pR115 derivative without *YPet*	This study
pR114	Mal11-D123A/E120A/E167A derivative of pRHA00L	This study
pR115	Mal11-D123A/E120A/E167Q derivative of pRHA00L	This study
pR116	Mal11-D123N/E120A/E167A derivative of pRHA00L	This study
pR117	Mal11-D123N/E120A/E167Q derivative of pRHA00L	This study
pR118	Mal11-D123N/E120Q/E167A derivative of pRHA00L	This study
pR230	pR118 derivative without *YPet*	This study
pR119	Mal11-D123N/E120Q/E167Q derivative of pRHA00L	This study
pRHM05A	Mal11-E146A derivative of pRHA00L	This study
pRHM05Q	Mal11-E146Q derivative of pRHA00L	This study
pRHM08A	Mal11-E150A derivative of pRHA00L	This study
pRHM11A	Mal11-E268A derivative of pRHA00L	This study
pRHM11Q	Mal11-E268Q derivative of pRHA00L	This study
pRHM12A	Mal11-D274A derivative of pRHA00L	This study
pRHM12N	Mal11-D274N derivative of pRHA00L	This study
pRHM14A	Mal11-E395A derivative of pRHA00L	This study
pRHM14Q	Mal11-E395Q derivative of pRHA00L	This study
pRHM15A	Mal11-D402A derivative of pRHA00L	This study
pRHM15N	Mal11-D402N derivative of pRHA00L	This study
pRHM16A	Mal11-E489A derivative of pRHA00L	This study
pRHM16Q	Mal11-E489Q derivative of pRHA00L	This study
pRHM17A	Mal11-E494A derivative of pRHA00L	This study
pRHM17Q	Mal11-E494Q derivative of pRHA00L	This study

pRHM18A	Mal11-D526A derivative of pRHA00L	This study
pRHM18N	Mal11-D526N derivative of pRHA00L	This study
pRHA27	Mal11-D551N derivative of pRHA00L	This study
pRHM19A	Mal11-E554A derivative of pRHA00L	This study
pRHM19Q	Mal11-E554Q derivative of pRHA00L	This study
pYES2-P_{ACT1}-pHluorin	*ACT1* promoter, pHluorin, *URA3*	[19]

Supplementary Table 4. Primers used in this study

Primer name	Sequence (5' to 3')	Description
5271	CAAGGAGAAAAAACCCCGGATTCTAGAACTAGTGGATCCCC CATGAAAAATATCATTTCATTGGTAAG	Fw primer for homologous recombination of Mal11 into pFB001
5272	GAATAATTCTTCACCTTTAGAACCTTGAAAATATAAATTTTCC CCTCCACATTTATCAGCTGCATTTAATTC	Rev primer for homologous recombination of Mal11 into pFB001
5273	GGAGGGGAAAATTTATATTTTCAAGGTTC	Fw primer for linearizing pFB001
5274	GGGGGATCCACTAGTTCTAGAATC	Rev primer for linearizing pFB001
5306	CCCTGGTTATGGAAGGTTATGCAACCGCACTACTGAGCG	D123A mutagenesis
5307	GTGCGCTCAGTAGTGCGGTTGCATAACCTTCCATAACC	D123A mutagenesis
5308	GTGTCTACTACCCTGGTTATGCAAGGTTATGATACCGCAC	E120Q mutagenesis
5309	GTAGTGCGGTATCATAACCTTGCATAACCAGGGTAGTAG	E120Q mutagenesis
5310	GAACGGGGAGGGTTCTTACCAAATTACTTCCCAATGGC	E150Q mutagenesis
5311	CCAATCTGCCATTGGGAAGTAATTTGGTAAGAACCCTCCC	E150Q mutagenesis
5312	ACATGTGTGTCCTTTGTGGTCAAATGATTGGTTTGCAAATCAC G	E167Q mutagenesis
5313	AAGTCGTGATTTGCAAACCAATCATTTGACCACAAAGGACAC	E167Q mutagenesis
5314	ACTTTAGCTTGGGTCATCATCAATCTGCCTGAGACAACTGG	D551N mutagenesis
5315	TTCTACCAGTTGTCTCAGGCAGATTGATGATGACCCAAGC	D551N mutagenesis
5435	CGCATCTGTGCGGTATTTC	Fw primer to amplify markers from pRS3xx plasmids
5436	GGCTTAACTATGCGGCATC	Rev primer to amplify markers from pRS3xx plasmids
5437	TGCACTCTCAGTACAATCTGCTC	Fw primer to amplify pRS3xx plasmids without amplifying the marker
5438	TGCGGTGTGAAATACCGC	Rev primer to amplify pRS3xx plasmids without amplifying the marker
5673	CCCTGGTTATGGAAGGTTATaacACCGCACTACTGAGCG	D123N mutagenesis
5674	CAGTGCGCTCAGTAGTGCGGTgttATAACCTTCCATAACC	D123N mutagenesis
5798	TCGGTACTTTGAACGGGgctGGTTCTTACGAAATTACTTCCC	E146A mutagenesis
5799	TTGGGAAGTAATTTCGTAAGAACCagcCCCGTTCAAAG	E146A mutagenesis
5800	TCGGTACTTTGAACGGGcaaGGTTCTTACGAAATTACTTCCC	E146Q mutagenesis
5801	TTGGGAAGTAATTTCGTAAGAACCttgCCCGTTCAAAG	E146Q mutagenesis
5802	GTGTCTACTACCCTGGTTATGgctGGTTATGATACCGCAC	E120A mutagenesis
5803	GTAGTGCGGTATCATAACCagcCATAACCAGGGTAGTAG	E120A mutagenesis
5804	GAACGGGGAGGGTTCTTACgctATTACTTCCCAATGGC	E150A mutagenesis

5805	CCAATCTGCCATTGGGAAGTAATagcGTAAGAACCCTCCC	E150A mutagenesis
5806	AACATGTGTGTCCTTTGTGGTgctATGATTGGTTTGC	E167A mutagenesis
5807	AAGTCGTGATTTGCAAACCAATCATagcACCACAAAGGAC	E167A mutagenesis
5808	TCGCCTCTGGTATTATGAAAAACTCACAAgctAATTTAGGGAA CTCCG	E268A mutagenesis
5809	TCGGAGTTCCCTAAATTagcTTGTGAGTTTTTCATAATACCAGA GG	E268A mutagenesis
5810	TCGCCTCTGGTATTATGAAAAACTCACAAcaaAATTTAGGGAA CTCCG	E268Q mutagenesis
5811	TCGGAGTTCCCTAAATTttgTTGTGAGTTTTTCATAATACCAGA GG	E268Q mutagenesis
5812	ACAAGAGAATTTAGGGAACTCCgctTTGGGCTATAAATTGCC	D274A mutagenesis
5813	AATGGCAATTTATAGCCCAAagcGGAGTTCCCTAAATTCTC	D274A mutagenesis
5814	ACAAGAGAATTTAGGGAACTCCaatTTGGGCTATAAATTGCC	D274N mutagenesis
5815	AATGGCAATTTATAGCCCAAattGGAGTTCCCTAAATTCTC	D274N mutagenesis
5818	ACTTGGTTACTCGACATATTTTTTTgctAGAGCAGGTATGGC	E395A mutagenesis
5819	TCGGTGGCCATACCTGCTCTagcAAAAAAATATGTCG	E395A mutagenesis
5820	ACTTGGTTACTCGACATATTTTTTTcaaAGAGCAGGTATGGC	E395Q mutagenesis
5821	TCGGTGGCCATACCTGCTCTttgAAAAAAATATGTCG	E395Q mutagenesis
5822	TGAAAGAGCAGGTATGGCCACCgctAAGGCGTTTAC	D402A mutagenesis
5823	AGAAAAAGTAAACGCCTTagcGGTGGCCATACCTGC	D402A mutagenesis
5824	TGAAAGAGCAGGTATGGCCACCaatAAGGCGTTTAC	D402N mutagenesis
5825	AGAAAAAGTAAACGCCTTattGGTGGCCATACCTGC	D402N mutagenesis
5826	AGTTGTTTACTGTATCGTTGCTgctATTCCATCAGCG	E489A mutagenesis
5827	AACTCCGCTGATGGAATagcAGCAACGATACAG	E489A mutagenesis
5828	AGTTGTTTACTGTATCGTTGCTcaaATTCCATCAGCG	E489Q mutagenesis
5829	AACTCCGCTGATGGAATttgAGCAACGATACAG	E489Q mutagenesis
5830	ATCGTTGCTGAAATTCCATCAGCGgctTTGAGAACTAAGAC	E494A mutagenesis
5831	ACTATAGTCTTAGTTCTCAAagcCGCTGATGGAATTTCAGC	E494A mutagenesis
5832	ATCGTTGCTGAAATTCCATCAGCGcaaTTGAGAACTAAGAC	E494Q mutagenesis
5833	ACTATAGTCTTAGTTCTCAAttgCGCTGATGGAATTTCAGC	E494Q mutagenesis
5834	TATATGCTAAACGTGAGCgctTGGAACTGGGGTGC	D526A mutagenesis
5835	TTTGGCACCCCAGTTCCAagcGCTCACGTTTAGC	D526A mutagenesis
5836	TATATGCTAAACGTGAGCaatTGGAACTGGGGTGC	D526N mutagenesis
5837	TTTGGCACCCCAGTTCCAattGCTCACGTTTAGC	D526N mutagenesis
5838	TTGGGTCATCATCGATCTGCCTgctACAACTGGTAGAACC	E554A mutagenesis
5839	TTCACTGAAGGTTCTACCAGTTGTagcAGGCAGATCG	E554A mutagenesis
5840	TTGGGTCATCATCGATCTGCCTcaaACAACTGGTAGAACC	E554Q mutagenesis
5841	TTCACTGAAGGTTCTACCAGTTGTttgAGGCAGATCG	E554Q mutagenesis
6017	AGTGTCTACTACCCTGGTTATGGCTGGTTATGCAACCGCACT ACTGAGCG	E120A/D123A mutagenesis
6018	AGTGTCTACTACCCTGGTTATGGCTGGTTATAACACCGCACT ACTGAGCG	E120A/D123N mutagenesis
6019	AGTGTCTACTACCCTGGTTATGCAAGGTTATGCAACCGCACT ACTGAGCG	E120Q/D123A mutagenesis

6020	AGTGTCTACTACCCTGGTTATGCAAGGTTATAACACCGCACT ACTGAGCG	E120Q/D123N mutagenesis
6021	ATACAGTGCGCTCAGTAGTGCGGTTGCATAACCAGCCATAAC CAGG	E120A/D123A mutagenesis
6022	ATACAGTGCGCTCAGTAGTGCGGTGTTATAACCAGCCATAAC CAGG	E120A/D123N mutagenesis
6023	ATACAGTGCGCTCAGTAGTGCGGTTGCATAACCTTGCATAAC CAGG	E120Q/D123A mutagenesis
6024	ATACAGTGCGCTCAGTAGTGCGGTGTTATAACCTTGCATAAC CAGG	E120Q/D123N mutagenesis
